# First description of the male of *Thaida chepu* Platnick, 1987 (Araneae, Austrochilidae) with micro-computed tomography of the palpal organ

**DOI:** 10.3897/zookeys.352.6021

**Published:** 2013-11-19

**Authors:** Peter Michalik, Martin J. Ramírez

**Affiliations:** 1 Allgemeine und Systematische Zoologie, Zoologisches Institut und Museum, Ernst-Moritz-Arndt-Universität, J.-S.-Bach-Str. 11/12, D-17489 Greifswald, Germany; 2 Research Associate, Division of Invertebrate Zoology, American Museum of Natural History, Central Park West at 79th Street, New York, NY 10024, USA; 3 Museo Argentino de Ciencias Naturales - CONICET, Buenos Aires, Argentina

**Keywords:** Taxonomy, micro-CT, spermophor, palp

## Abstract

The male of the austrochilid spider *Thaida chepu* Platnick, 1987 is described for the first time. We analyzed the internal anatomy of the palpal organ by using micro-computed tomography to investigate the spermophor as well as the muscles and tendons in the cymbium and tibia in detail. As shown by our data, muscles 29 and 30 originate in the tibia and continue with tendons to the base of the bulb, which resembles the ancestral organization for the male palp of spiders; this condition has not been described for Araneomorphae until now. The 3D reconstruction of the spermophor confirms recent interpretations of the male palp sclerites within Austrochilidae.

## Introduction

The family Austrochilidae consists of three genera with a very peculiar distribution. Whereas the genera *Austrochilus* Gertsch & Zapfe, 1955 (6 species) and *Thaida* Karsch, 1880 (2 species) are endemic to the forests of Central and Southern Chile and adjacent Argentina, the monotypic genus *Hickmania* Gertsch, 1958 is endemic to Tasmania (Forster et al. 1987). The Austrochilidae, which together with the family Gradungulidae comprise the superfamily Austrochiloidea, are of high interest for spider systematics since it is ambiguously placed among the early derivative taxa of Araneomorphae (e.g., Griswold et al. 2005). Thus, detailed knowledge of taxon-specific structures such as the genitalia is highly valuable not only for species determination but also for a better understanding of interrelationships among the Araneomorphae. In the present study, we describe the male of *Thaida chepu* Platnick, 1987 for the first time. Furthermore, we studied the internal anatomy of the male palpal organ using X-ray microtomography (micro-CT) in order to reconstruct the spermophore (sperm duct) and position of the embolus, which was debated in former studies (see Griswold et al. 2005: 17).

## Material and methods

**Figure 1–4.**
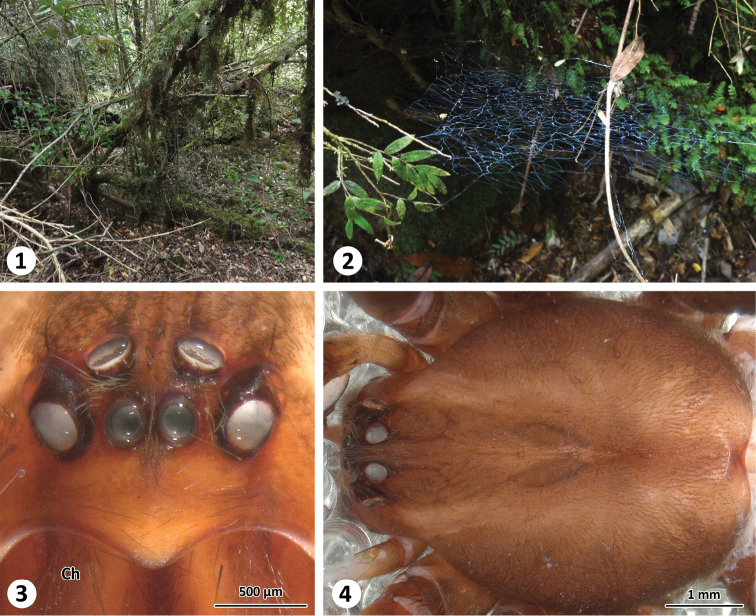
Habitat (**1**), web (**2**) and somatic characters (frontal (**3**) and dorsal (**4**) view of prosoma) of *Thaida chepu*.

We collected a male of *Thaida chepu* close to the type locality in a wet lowland mixed forest at Lago Huillinco (Chiloé, Chile) (Fig. 1). The material was examined and documented (extended focal range images) in 80% ethanol using a Zeiss Discovery V20 stereo microscope with a Zeiss MCr camera. Editing of images to adjust brightness, contrast and color was performed using Adobe Photoshop CS4. Measurements (given in millimeters) were obtained from digital images using the IntMess module in the program Zeiss AxioVision 4.8 (Carl Zeiss MicroImaging GmbH, Göttingen, Germany). The style of the description is based on Forster et al. (1987) and Grismado et al. (2003).

For the micro-CT analyses of the male palp, the sample was dehydrated in graded ethanol and stained with a 1% iodine solution for 12 hours. After washing in pure ethanol, the sample was scanned in ethanol with an Xradia MicroXCT-200 X-ray imaging system (Carl Zeiss X-ray Microscopy Inc., Pleasanton, USA) at 40 kV and 8 W using phase contrast (4.0 scintillator-objective lens unit, 15 s exposure time, 4.15 µm pixel size). The obtained data were processed using the 3D analysis software AMIRA v. 5.4.2 (Visage Imaging, Berlin, Germany). Virtual reconstruction of the spermophore was performed by delineation in each section (segmentation) and a smooth surface was computed using the surface editor. The image stack is stored in MorphDBase under creative commons attribution (CC-BY; ID: P_Michalik_20130729-M-4.1; https://www.morphdbase.de?P_Michalik_20130729-M-4.1).

## Abbreviations

ALE anterior lateral eye

AME anterior median eye

bH basal hematodocha

Cb cymbium

Ch chelicera

E embolus

HSt hook of subtegulum

m29 muscle 29

m30 muscle 30

mA median apophysis

mH median hematodocha

MOQ median ocular quadrangle

PLE posterior lateral eye

PME posterior median eye

PSt process of subtegulum

S spermophor

St subtegulum

Te tegulum

tm29 tendon of muscle 29

tm30 tendon of muscle 30

ZIMG Zoologisches Institut und Museum Greifswald (Germany)

## Taxonomy

### Family Austrochilidae Zapfe 1955, Subfamily Austrochilinae Zapfe 1955, Genus *Thaida* Karsch 1880

#### Thaida chepu

Platnick, 1987

http://species-id.net/wiki/Thaida_chepu

##### Material examined.

CHILE: Region de Los Lagos (X), Chiloé province, Isla de Chiloé, Lago Huillinco, N margin, 4.6 km (air) ESE Cucao, 42.64117°S, 74.04763°W (GPS, ±100m), elev. 12 m (MJR-loc-86), 16 February 2012, 1 male, coll. K. Huckstorf, M. Izquierdo, P. Michalik, M. J. Ramirez, C. S. Wirkner (ZIMG II/28126).

##### Diagnosis.

**Figure 5–10.**
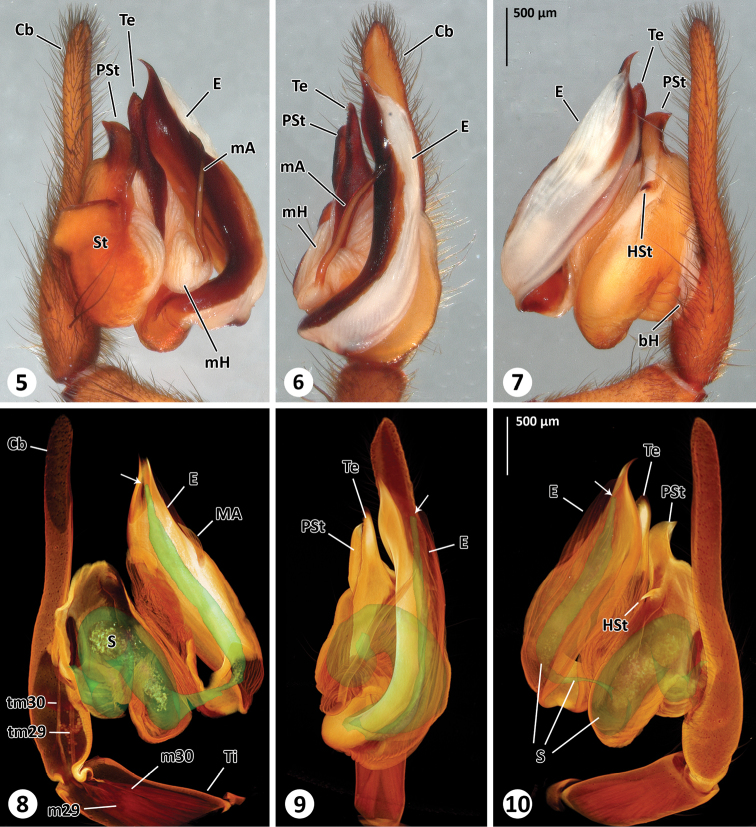
Left male palp of *Thaida chepu*. **5–7** extended focal plane images of male palp in prolateral (**5**), ventral (**6**) and retrolateral (**7**) view **8–10** surface model of the spermophor superimposed on the volume rendering of the male palp to illustrate dimension and shape of the spermophor. The views correspond to Figs **5–7**. The cymbium, subtegulum and tegulum are (partly) removed in Fig. 8 to show tendons and muscles. The arrows point to the opening of the embolus.

Similar to *Thaida peculiaris* by the clypeus about three times the diameter of the anterior median eyes (Fig. 3); males distinguished from *Thaida peculiaris* by the copulatory bulb, which has the median apophysis longer than the embolus (Figs 5–10; about half the length of the embolus in *Thaida peculiaris*, Forster et al. 1987, figs 155–157), a stout and curved process of the subtegulum (slender and straight in *Thaida peculiaris*, Forster et al. 1987, figs 155–157), and the bent embolus with a curved tip (embolus of *Thaida peculiaris* without distinct tip, Forster et al. 1987, figs 155–157). Diagnosis of the female in Forster et al. (1987).

##### Description.

**Male (ZIMG II/28126).** Carapace 5.59 long and 4.38 wide; clypeal height 0.68 (in the middle about three times AME diameter in height; Fig. 3); coloration as depicted in Fig. 4. Eye sizes and interdistances: AME 0.24, ALE 0.31, PME 0.25, PLE 0.30; AME-AME 0.05, AME-ALE 0.15, PME-PME 0.26, PME-PLE 0.22, ALE-PLE 0.06; MOQ length 0.77, median ocular quadrangle width 0.78. Spination: femora: I d 1-0-1, p 2-3-3, r 2-3-3; II d 1-2-1, p 2-2-2, r 2-2-2; III d 1-2-1, p 1-2-2, r 2-2-2; IV d 2-2-1, p 0-1-2, r 0-1-3; tibiae: I p 2-3-2, v 3-3-4, r 2-2-2; II p 2-2-2, v 3-4-3, r 1-1-2; III d 1-0-1, p 0-2-2, v 1-1-4, r 0-2-1; IV missing; metatarsus: I p 3-1-2, v 1-1-1, r 2-2-2; II d 0-1-2, p 1-2-2, v 0-1-2, r 1-1-0; III p 1-1-2, v 2-3-2, r 2-2-1; IV missing. Palp (Figs 5-10): cymbium slender, median apophysis slender and spine-shaped with serrated tip, embolus broad with twisted, membranous flange and slit-like opening, membranous spermophor as depicted in Figs 8–10, m29 and m30 originating in tibia. The 3D reconstruction revealed that the spermophor fills most of the subtegulum and is flattened and thin within the embolus (Video 1). Abdomen missing.

**Video 1.** Surface model of the spermophor superimposed on the volume rendering of the male palp. Video available for download in full resolution from http://www.pensoft.net/J_FILES/1/articles/6021/export.php_files/Michalik_Ramirez_Video_1.avi.

##### Appendages measurements:

**Table:** 

	I	II	III	IV	Palp
Femur	8.82	7.67	5.95	7.3	2.99
Patella	2.02	1.83	1.54	1.69	0.92
Tibia	9.91	7.55	4.63	missing	1.43
Metatarsus	9.40	7.60	5.39	missing	-
Tarsus	3.68	3.04	2.17	missing	3.27
**Total**	33.84	27.71	19.69		8.62

##### Natural history.

The webs of *Thaida chepu* are very similar to those described for *Thaida peculiaris* Karsch 1880 and *Austrochilusforsteri* Grismado, Lopardo & Platnick, 2003 by Lopardo et al. (2004) (Fig. 2).

## Discussion

**Figure 11.**
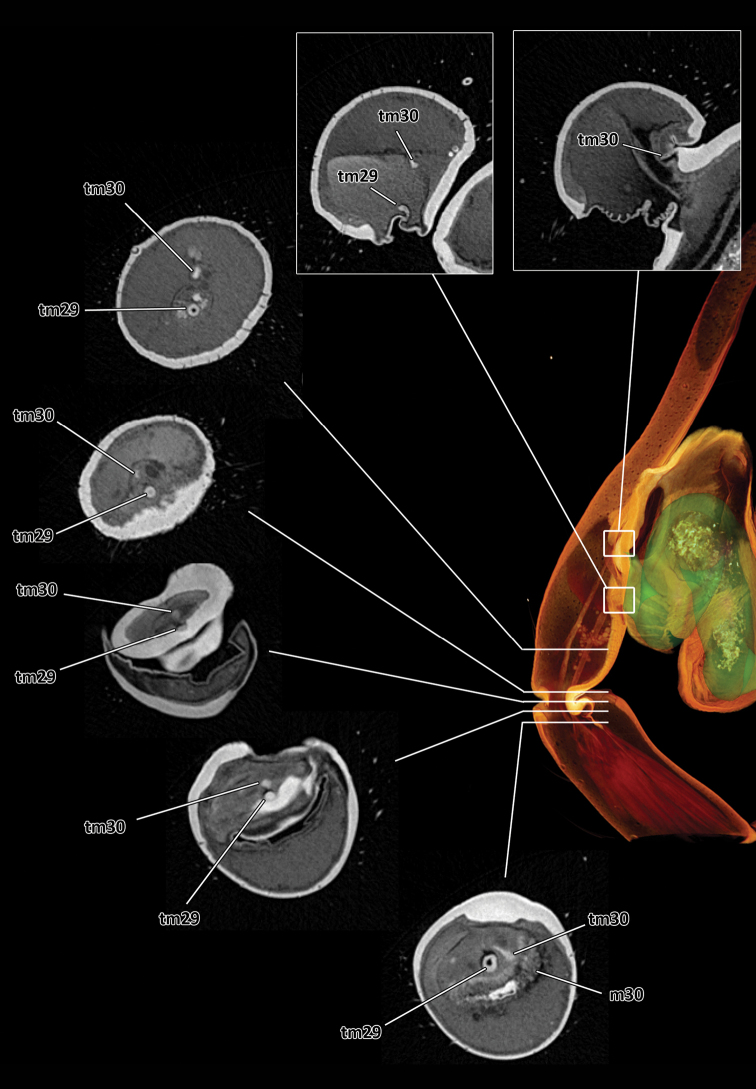
Series of virtual cross sections through the left male palp of *Thaida chepu* showing the course of the two tendons.

Based on the micro-CT data and manual segmentation of the spermophor we confirm the interpretation of the male palp sclerites especially with regard to the position of the embolus given by Griswold et al. (2005). The spermophor of austrochilines is membranous, with thin cuticle, not evident without preparation (e.g., micro-CT or clearing by clove oil; see also Huber 2004). Moreover and in contrast to the findings of Huber (2004) on *Thaida peculiaris*, our micro-CT analysis revealed that no muscles originate in the cymbium (tarsus) of *Thaida chepu*. Instead, the muscles 29 and 30 originate in the tibia and are connected by tendons with the bulbal sclerites (Figs 8, 11) – an organization only known from the basal spider genera *Liphistius* Schiödte, 1849 and *Atypus* Latreille, 1804 (Huber 2004). Preliminary micro-CT analyses of the palp of the sister group Hickmaniinae (*Hickmania troglodytes*) (Lipke, personal communication), histological sections as well as micro-CT analyses of representatives of Gradungulidae (Huber 1994; Michalik et al. 2013) and *Hypochilus* (Hypochilidae) (Huber 1994) revealed that the muscle 30 originates in the cymbium as typical for araneomorph spiders (Huber 2004). This is especially important since austrochilids are key taxa that might reveal important information to interpret the transition from muscular to hydraulically-controlled copulatory organs (Huber 2004), and towards the evolution of higher Araneomorphae (i.e. Haplogynae and Entelgynae).

As shown here micro-CT data can be used for precise and transparent descriptions (for details on the method and data handling see Faulwetter et al. 2013) as well as revealing internal anatomical characters useful for spider taxonomy, systematics and evolutionary/functional morphology.

## Supplementary Material

XML Treatment for Thaida chepu
